# Nature of fatty acids in high fat diets differentially delineates obesity-linked metabolic syndrome components in male and female C57BL/6J mice

**DOI:** 10.1186/1758-5996-3-34

**Published:** 2011-12-14

**Authors:** Souhad El Akoum, Vikie Lamontagne, Isabelle Cloutier, Jean-François Tanguay

**Affiliations:** 1 Montreal Heart Institute, 5000 Belanger, Montréal (QC) H1T 1C8, Canada; 2 Département de Sciences Biomédicales, Faculté de Médecine, Université de Montréal, 2900 boulevard Edouard-Montpetit, Montréal (QC) H3T 1J4, Canada

**Keywords:** Adipocyte, Adipokines, Diabetes, High Fat Diet, Metabolic disorders, Obesity, Type 2 Diabetes

## Abstract

**Background:**

Adverse effects of high-fat diets (HFD) on metabolic homeostasis are linked to adipose tissue dysfunction. The goal of this study was to examine the effect of the HFD nature on adipose tissue activity, metabolic disturbances and glucose homeostasis alterations in male mice compared with female mice.

**Methods:**

C57BL/6J mice were fed either a chow diet or HFD including vegetal (VD) or animal (AD) fat. Body weight, plasmatic parameters and adipose tissue mRNA expression levels of key genes were evaluated after 20 weeks of HFD feeding.

**Results:**

HFD-fed mice were significantly heavier than control at the end of the protocol. Greater abdominal visceral fat accumulation was observed in mice fed with AD compared to those fed a chow diet or VD. Correlated with weight gain, leptin levels in systemic circulation were increased in HFD-fed mice in both sexes with a significant higher level in AD group compared to VD group. Circulating adiponectin levels as well as adipose tissue mRNA expression levels were significantly decreased in HFD-fed male mice. Although its plasma levels remained unchanged in females, adiponectin mRNA levels were significantly reduced in adipose tissue of both HFD-fed groups with a more marked decrease in AD group compared to VD group. Only HFD-fed male mice were diabetic with increased fasting glycaemia. On the other hand, insulin levels were only increased in AD-fed group in both sexes associated with increased resistin levels. VD did not induce any apparent metabolic alteration in females despite the increased weight gain. Peroxisome Proliferator-Activated Receptors gamma-2 (PPARγ2) and estrogen receptor alpha (ERα) mRNA expression levels in adipose tissue were decreased up to 70% in HFD-fed mice but were more markedly reduced in male mice as compared with female mice.

**Conclusions:**

The nature of dietary fat determines the extent of metabolic alterations reflected in adipocytes through modifications in the pattern of adipokines secretion and modulation of key genes mRNA expression. Compared with males, female mice demonstrate higher capacity in controlling glucose homeostasis in response to 20 weeks HFD feeding. Our data suggest gender specific interactions between the diet's fatty acid source, the adipocyte-secreted proteins and metabolic disorders.

## Background

Obesity is defined as an increase of adipose tissue mass in the body and its accumulation in peripheral organs that leads to metabolic abnormalities such as type 2 diabetes (T2D), insulin resistance and hyperlepidemia [1,2]. Obesity is thus a worldwide healthcare problem increasing morbid-mortality [2].

The visceral adipose tissue plays an important role in the regulation of postprandial lipid and glucose systemic homeostasis by targeting essential organs (adipose tissue, muscle, etc.) and systems (neuroendocrine axis, etc.) [3-6]. It is considered as a secretory gland source of several bioactive peptides called adipokines [6]. Thus, adipose tissue becomes a major protagonist of metabolic alterations triggered by lipid over-accumulation in their cytoplasmic droplet which leads to adipocytes function disorders [7,8]. An alteration in the adipokine secretion profile leads to insulin resistance, glucose intolerance and lipid metabolic disturbances [8-10].

Consumption of High-fat diets (HFD) is a central risk factor for metabolic disorders linked to obesity [9,11]. Adverse effects of HFD on metabolic homeostasis are linked to adipose tissue physiology and are highly influenced by gender [12,13]. The imbalance between caloric intake and energy expenditure leads to hyperplasia and hypertrophy of adipocytes depending on the type as much as the amount of dietary lipids [14,15].

Numerous factors regulate the adipose tissue activity including adipocyte-specific genes such as peroxisome proliferator-activated receptors gamma (PPARγ) [16]. Dietary fatty acids (FA) and their derivatives are described as PPARγ ligands that trigger physiological responses such as adipogenesis and adipokine secretion. Thus, HFD-inducing metabolic disorders act via PPARγ to induce different levels of systemic homeostatic remodelling.

Appreciating the pathogenesis of HFD-induced metabolic disorders requires a thorough knowledge of adipose tissue physiology and the regulation of adipokines secretion and action including the role of gender in response to these parameters.

Thus, significant progress has been made in our understanding of the relation between HFD feeding and adipose tissue dysfunction. However, experimental evidence for HFD-mediating metabolic alterations remains to be elucidated. Therefore, we propose a possible role for HFDs' fat nature on the establishment of metabolic disorders. Herein, we evaluated the impact of FA nature on the development of metabolic alterations through modulation of adipose tissue activity and secretion profiles. We also determined gender-specific impact on the kinetic of metabolic disorders progression in response to these diets.

## Methods

### Experimental protocol

The animal protocol was approved by the Animal Care and Use Committee of the Montreal Heart Institute conforming to the Guide for the Care and Use of Laboratory Animals published by the US National Institutes of Health (NIH Publication No. 85-23, revised 1996). Three groups of 10 male and 10 female C57BL/6J mice (Jackson Laboratory, Bar Harbor, MN, USA) were included in this study at 5 weeks of age. Each group was fed either standard diet (SD) used as control (6% fat, 57% sucrose) or one of the two low cholesterol HFD (34.9% fat, 26.3% sucrose) (detailed composition presented in Table 1). These latter were iso-caloric but differed in fat nature: VD was composed of soy and cotton oil while AD was composed of lard. Weight gain was monitored during the 20 weeks of protocol and daily food consumption was calculated by subtracting the residual quantity from the supplemented food quantity each day. Energy intake was calculated on the basis of 3.8 kcal/g for the SD and 5.2 kcal/g for both HFD.

**Table 1:** Analysis of diets compositions

Supplier Cat#	ResearchDiet D06061202	ResearchDiet D12492	Harlan-Teklad 2018
**Components**	**VD**	**AD**	**SD**

**Sucrose**	26,3%	26,3%	57%

**Protein**	26,2%	26,2%	18,9%

**Lipid**	34,9%	34,9%	6%

**FA composition:**	**g/100 g of diet**		

C14 Myristic	0,2	0,9	0,006

C14:1 Myristoleic	0	0,5	0

C16 Palmitic	13,0	21,8	0,764

C16:1 Palmitoleic	0,2	3,8	0

C18 Stearic	10,2	12,4	0,15

C18:1 Oleic	62,0	39,3	1,26

C18:2 Linoleic	12,9	12,8	3,13

C18:3 Linolenic	0,7	1,6	0,28

C20 Arachidic	0,3	0	0,01

C20:4 Arachidonic	0	1,7	0

C22 Behenic	0,3	0	0,003

C24 Lignoceric	0,1	0	0

Cholesterol	0,035	0,030	

**FA proportions:**	**Percentage of total FA**		

Saturated FA	24,2%	37,0%	16,96%

Mono-unsaturated FA	62,2%	46,0%	22,71%

Poly-unsaturated FA	13,6%	17,0%	60,33%

provided by the manufacturer (Research Diets, Inc).

### Intra-Peritoneal glucose tolerance test

The Intra-Peritoneal Glucose Tolerance Test (IPGTT) was performed after 20 weeks of diet following overnight (18 hr) fasting. After measuring the fasting glycæmia using an Accu-Check^® ^(Roche Diagnostics, Laval, QC, Canada) glucometer, mice received an intra-peritoneal injection of glucose solution (2 g/kg). Subsequent measures of plasma glucose levels were performed 30, 60, 90, 120 and 180 minutes post-injection. The area under the glucose tolerance curve (AUC) was measured to evaluate mice glucose tolerance.

### Physiological analyses

Non-invasive measurement of mice cardiac function was performed by Doppler echocardiography under anesthesia (isoflurane 2.5%). The velocities of the early mitral flow (E) over the late mitral flow (A) were measured with a Doppler Signal Processing Workstation (GE-Ultrasound System). An increase in the E/A ratio, with restrictive aspect of transmitral flow (E/A > 2) indicated a diastolic dysfunction with increased LV filling pressure. To calculate ventricular mass, left ventricular (LV) tele-diastolic diameter, posterior wall and inter-ventricular septum thickness were measured.

### Biochemical analyses

At sacrifice, after an overnight fast (18 hr), blood samples were gathered by cardiac exsanguinations and plasma was collected and stored at -20°C until analysis. Plasma adiponectin and leptin concentrations were measured using mouse ELISA kits (ALPCO, Salem, NH, USA) according to the manufacturer's instructions. Plasma insulin, resistin and TNFα levels were analyzed using mouse ELISA kits (AssayPro, St. Charles, MO, USA) according to the manufacturer's instructions. All measurements were analyzed in duplicate for at least 8 animals per group. The circulating concentrations of FA in mice serum were evaluated using ADIFAB free FA indicator (Invitrogen, Burlington, ON, Canada).

### Homeostasis model assessment of insulin resistance (HOMA-IR)

Insulin resistance was assessed by calculation of HOMA-IR using glucose and insulin concentrations obtained after overnight fasting (18 hr), using the following formula:

$$\text{HOMA}-\text{IR }\left(\text{mmol}∕\text{L }\times \mu \;\;\text{U}∕\text{ml}\right)=\text{ fasting glucose }\left(\text{mM}\right)\times \text{ fasting insulin }\left(\mu \;\;\text{U}∕\text{ml}\right)∕\text{22}.\text{5}$$

HOMA-IR is known to be correlated with the insulin sensitivity evaluated by the euglycemic hyperinsulinemic clamp [17,18].

### Quantitative real-time PCR (Q-PCR)

At sacrifice, mouse adipose tissue was removed from the abdominal visceral region weighed to evaluate its accumulation level, and then frozen until mRNA extraction. Total RNA was isolated from approximately 30 mg of frozen white adipose tissue of the abdominal visceral region using Qiazol reagent according to the manufacturer's instructions (Qiagen, Toronto, ON, Canada). Single-strand cDNA was synthesized according to the procedure in the iScript cDNA Synthesis Kit manual (Bio-Rad Laboratories, Montreal, QC, Canada). Q-PCR reactions were carried out using the Brilliant-II SYBR^® ^Green Master-Mix (Stratagene, Mississauga, ON, Canada) and specific primers:

• Adiponectin primers (#GenBank: NM_009605.4):

○ Fwd 5'-GAA-TCA-TTA-TGA-CGG-CAG-CA-3'

○ Rev 5'-TCA-TGT-ACA-CCG-TGA-TGT-GGT-A-3'

• Leptin primers (#GenBank: NM_008493.3):

○ Fwd 5'-GAC-ATT-TCA-CAC-AGG-CAG-TCG-3'

○ Rev 5'-GCA-AGC-TGG-TGA-GGA-TCT-GT-3'

• TNFα primers (#GenBank: NM_013693.2):

○ Fwd 5'-CAT-CTT-CTC-AAA-ATT-CGA-GTG-ACA-A-3'

○ Rev 5'-TGG-GAG-TAG-ACA-AGG-TAC-AAC-CC-3'

• PPARγ2 primers (#GenBank: NM_011146.3):

○ Fwd 5'-AGC-ATG-GTG-CCT-TCG-CTG-AT-3'

○ Rev 5'-GGT-GGA-GAT-GCA-GGT-TCT-AC-3'

• ERα primers (#GenBank: NM_007956.4):

○ Fwd 5'-TCT-GAC-AAT-CGA-CGC-CAG-AA-3'

○ Rev 5'-TAA-CAC-TTG-CGC-AGC-CGA-CA-3'

The mRNA levels were normalized to Cyclophilin-A expression levels (Fwd 5'-CCG-ATG-ACG-AGC-CCT-TGG-3'; Rev 5'-GCC-GCC-AGT-GCC-ATT-ATG-3'). The targeted and referenced genes were amplified in duplicate in the same run using the Mx3000P Q-PCR System (Stratagene).

The relative quantification of target genes was determined using the MxProTM Q-PCR software version 3.00 (Strategene). Briefly, Ct average of each duplicate was calculated for each gene and Cyclophilin-A and the ΔCT (CTgene - CTCyclo-A) was determined. The control adipose tissue sample was chosen as a reference sample and set as 100% of gene quantity. Finally, the mRNA abundance of other samples to the mRNA abundance of the control adipose tissue was calculated with use of the formula 2^-ΔΔCT^.

### Statistical Analyses

All statistical analyses were performed separately for males and females.

Data are presented as mean ± standard deviation for continuous variables. Repeated measures analysis of covariance (ANCOVA) model was used to study weight gain across the study time and between groups (SD, VD and AD groups) adjusted for baseline weight value. The group × time interaction was also included in the ANCOVA model and comparisons between groups at a given time point were undertaken only in the presence of a significant group × time interaction. Otherwise, global conclusions were drawn based on the main time and group effects of the model.

Repeated measures analysis of variance (ANOVA) models were used to study the glycemic parameters across the IPGTT time (0, 30, 60, 90, 120 and 180 min) and between groups. Models with time, group and group × time interaction as independent variables were used. Comparisons between groups at a given time-point were undertaken only in the presence of significant group × time interaction. Otherwise, global conclusions were drawn based on the main time and group effects of the model.

The insulin, adiponectin, leptin, TNFα and FA levels were compared between groups (SD, VD and AD groups) using an ANOVA model.

In addition, the relationships among weight gain, cardiac parameters and metabolic parameters were investigated using Pearson or Spearman correlations according to the nature of the data distribution.

All analyses were done using SAS version 9.1 (SAS Institute Inc., Cary, NC, USA) and conducted at the 0.05 significance level.

## Results

### Weight gain and adipose tissue mass

**Figure 1:**
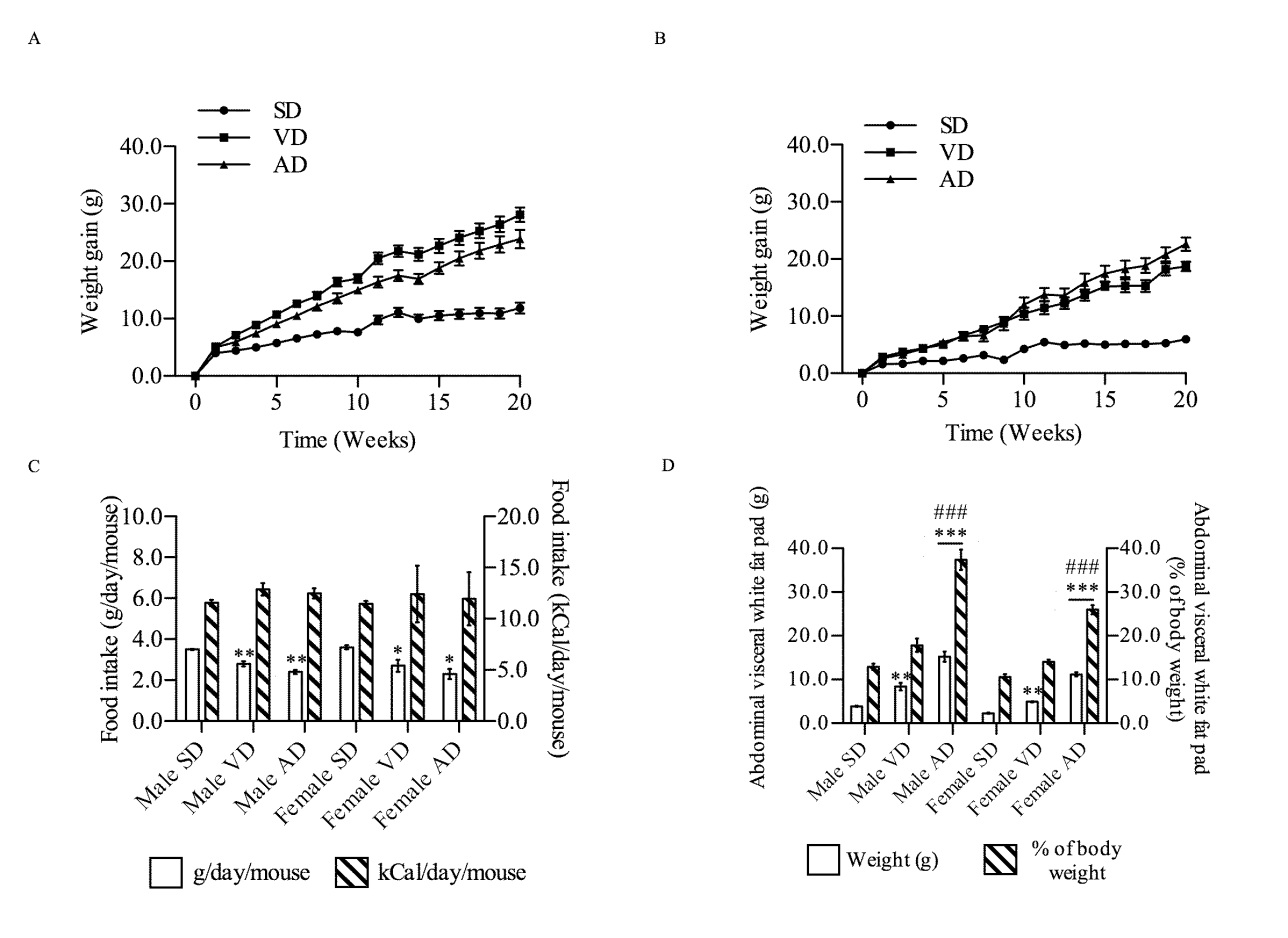
**Kinetic of weight gain in male (A) and female (B) mice (n = 10)**. The mean cumulative food intake (C) was evaluated during the protocol in g/day/mouse (white column) and kCal/day/mouse (hatched column). White fat pad mass (D) is expressed as the weight of adipose tissue extracted from the abdominal visceral region of each mouse (white column) and transposed in percentage of total body weight (hatched column) at the time of the sacrifice (n = 9). * P < 0.05, ** P < 0.01, *** P < 0.001 vs. SD; ### P < 0.001 vs. VD.

After 20 weeks of diet, the mean weight in SD group had increased by 64% in males compared to their weight at the beginning of the protocol (Figure 1.A). A more pronounced increase was observed in VD (150%, P < 0.001) and AD groups (144%, P < 0.001). Comparable results were obtained in females fed with VD (116%) and AD (137%) compared to the SD group (39%, P < 0.001) (Figure 1.B). Despite the differences in weight gain, the daily food intake (kCal/day/mouse) was similar in both sexes notwithstanding the different diets (Figure 1.C).

However, the weight gain observed in HFD groups did not reflect comparable amounts of adipose tissue accumulation in the abdominal visceral region (Figure 1.D). We showed that male and female mice under SD accumulated 3.9 g and 2.3 g respectively after 20 weeks of diet. For the same feeding time, considerable increase was seen in adipose tissue mass in male and female mice under VD (8.4 g and 4.9 g respectively) and AD (15.2 g and 11.2 g respectively) compared to SD group (P < 0.01). In the HFD group, for a similar weight gain, abdominal-visceral adipose tissue constituted 37.4% of total body weight in male mice under AD; a 2-fold increase as compared to the VD group (17.8%, P < 0.001) and a 3-fold increase in comparison with the SD group (12.9%, P < 0.001). Similar results were obtained in females (10.6% for SD group; 14.0% for VD group; 26.0% for AD group) (P < 0.001).

### Glycemic parameters

**Figure 2:**
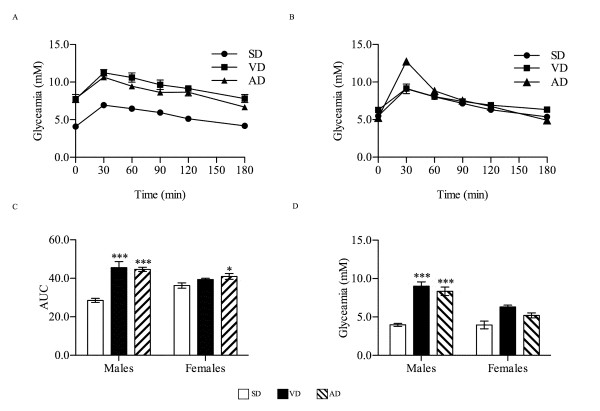
**Glycemic parameters**. After overnight fasting, IPGTT challenge was carried at the end of the protocol in males (A) and females (B) (n = 9). From these curves, AUC were calculated to estimate glucose tolerance in corresponding mice (C). Fasting glycæmia (D) was measured at the beginning of the IPGTT. * P < 0.05, *** P < 0.001 vs. SD.

The IPGTT showed a glycæmia peak 30 min post-challenge in the SD male group (Figure 2.A). Glucose elimination followed first order kinetic curve thereafter to reach basal level after 180 minutes. Both HFD groups showed an equivalent glucose elimination kinetic curve with higher blood glucose levels throughout the duration of the test. This led to an increased AUC in these groups (45.5 for VD, 44.6 for AD) compared to SD (28.6, P < 0.001) (Figure 2.C). In contrast to male groups, female groups responded such that only AD showed an increased glycæmia 30 min post-challenge (Figure 2.B) with slight glucose intolerance reflected by an increased AUC (41.0) compared to SD (36.3, P < 0.01) (Figure 2.C). AUC of the VD female group remained unchanged (39.4).

Both HFD male groups had an increased fasting glycæmia (Figure 2.D) and are therefore considered diabetic in addition to being glucose intolerant. In contrast no change in fasting glycæmia was observed in the female groups.

### Biochemical blood analysis

**Figure 3:**
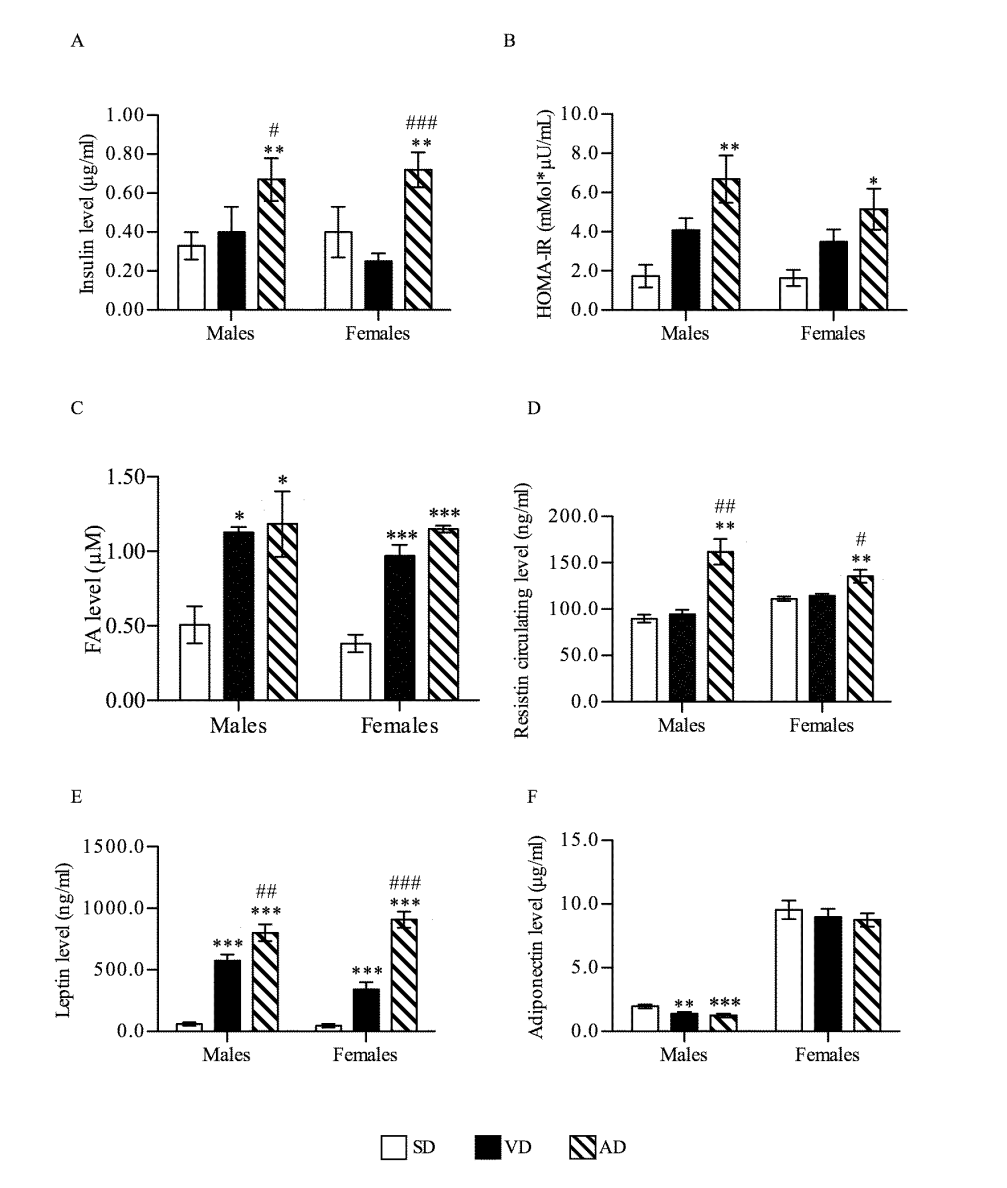
**Circulating levels of insulin (A), FA (C), resistin (D) leptin (E) and adiponectin (F) circulating levels were evaluated at the sacrifice after overnight fasting in all mice groups (n = 8)**. HOMA-IR (B) was calculated according to fasting glycæmia and insulinæmia using the following formula: HOMA-IR (mmol/L × μU/ml) = fasting glucose (mM) × fasting insulin (μU/ml)/22.5. * P < 0.05, ** P < 0.01, *** P < 0.001 vs. SD; # P < 0.05, ## P < 0.01, ### P < 0.001 vs. VD.

Insulin concentration was significantly increased in AD male (0.67 μg/ml) and female (0.72 μg/ml) groups compared to SD group (0.33 μg/ml for male, P < 0.01; 0.40 μg/ml for female, P < 0.01), and VD group (0.40 μg/ml for male, P < 0.05; 0.25 μg/ml for female, (P < 0.001) (Figure 3.A). These results emphasize the T2D state of AD male mice shown to have developed glucose intolerance and increased fasting glycæmia. Furthermore, the HOMA-IR factor, reflecting the insulin resistance, was also significantly increased in the AD group (Figure 3.B).

Circulating FA levels were highly increased in HFD groups for both genders (P < 0.01) (Figure 3.C). HFD raised circulating FA by 130% in males (1.13 μM for VD, 1.18 μM for AD) and up to 200% in females (0.97 μM for VD, 1.15 μM for AD) compared to their respective control (0.50 μM for male, 0.38 μM for female), without significant differences between HFD groups.

Adipokine secretion profiles in mice blood were evaluated. A marked increase in resistin levels (P < 0.05) was measured in AD groups (161.9 ng/ml for males and 135.6 ng/ml for females) compared to SD and VD groups (P < 0.05) (Figure 3.D). Interestingly, resistin levels were significantly higher in females under SD (111.2 ng/ml) and VD (114.4 ng/ml) compared to the corresponding male groups (89.8 ng/ml; 94.6 ng/ml respectively) (P < 0.05).

High levels of leptin were found in both VD (575.0 ng/ml) and AD (801.1 ng/ml) male groups compared to SD group (57.8 ng/ml), with a 40% higher level in AD vs. VD groups (P < 0.001) (Figure 3.E). A comparable hyperleptinemia profile was detected in females under HFD compared to the control group (42.4 ng/ml) with a more marked difference (166%) between VD (341.1 ng/ml) and AD (908.0 ng/ml) groups (P < 0.001).

On the other hand, circulating adiponectin was decreased in male mice of VD (1.4 μg/ml) and AD (1.2 μg/ml) groups compared to control (2.0 μg/ml, P < 0.001), an effect not observed among female groups (Figure 2.F). Moreover, females presented a 5 to 7-fold higher adiponectinemia than corresponding male groups (P < 0.001).

Finally, TNFα circulating levels remained unchanged in males and females of the different diet groups (data not shown).

### Adipokine mRNA expression levels

Adipokine mRNA expression levels were evaluated in the adipose tissue obtained from the abdominal visceral region.

**Figure 4:**
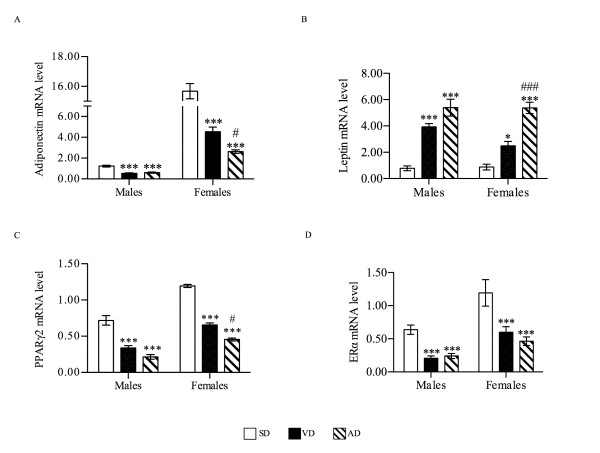
**Expression profile of adipokines genes**. Adiponectin (A), leptin (B), PPARγ2 (C) and ERα (D) mRNA expression levels in abdominal visceral white adipose tissue extracted from all groups (n = 8) were evaluated by Q-PCR. mRNA expression level was reported as a ratio over the expression level of the reference gene Cyclophilin-A. * P < 0.05, ** P < 0.01, *** P < 0.001 vs. SD; # P < 0.05, ### P < 0.001 vs. VD.

A marked decrease in adiponectin mRNA levels (P < 0.001) was measured in white adipose tissue of VD male (0.54) and female (4.53) groups and AD male (0.61) and female (2.84) groups compared to control (1.24 for males and 15.67 for females) (Figure 4.A). This decrease was more pronounced in the AD female group compared to the VD group (P < 0.05). Overall, adiponectin mRNA levels in HFD-fed females remained 5 to 12 times higher than levels in HFD-fed males.

As seen in blood protein levels, leptin mRNA levels were also significantly increased in white adipose tissue of HFD male mice (3.93 for VD and 5.39 for AD group) compared to control (0.78) (P < 0.001) with no difference between the two HFD groups (Figure 4.B). In females, on the other hand, VD increased leptin mRNA levels slightly in visceral adipose tissue (2.49) compared to control (0.88) (P < 0.05) though AD showed a more marked increase of leptin mRNA (5.37) compared to SD (P < 0.001) and VD (P < 0.001).

We then evaluated mRNA expression levels of PPARγ2, nuclear receptors implicated in adipokine transcription control. HFD significantly reduced PPARγ2 mRNA expression in males fed with VD (0.34) and AD (0.21), and in females fed with VD (0.65) and AD (0.46) compared to control (P < 0.001) (Figure 4.C). However, PPARγ2 expression levels remained up to 2-times superior in the abdominal visceral adipose tissue of females compared to the corresponding male groups.

Since previous reports showed crosstalk between estrogens receptors (ER) and PPARγ regulatory pathways [16], we evaluated the mRNA expression levels of its alpha subtype (ERα). ERα mRNA expression was down-regulated by 70% in male HFD groups and 50% in female HFD groups compared to control (P < 0.001) (Figure 4.D). No diet-specific difference was detected between HFD groups but ERα mRNA expression was 3 times higher in females compared to the corresponding male groups.

Finally, TNFα mRNA expression levels were unchanged in white adipose tissue in female groups compared to control, but were significantly increased in males fed with AD (24.90) compared to control (2.25) (P < 0.01), but not in VD (9.20) (Additional file 1: Figure S1).

### Cardiac parameters

To investigate cardiac consequences of HFD, non-invasive measurements of cardiac parameters were performed by Doppler echography. Cardiac-diastolic dysfunction characterized by an elevation of the ratio of the early mitral flow (E) over the late mitral flow (A) and LV hypertrophy was observed in some HFD groups. Thus, a significant increase in E/A ratio by 63% and 59% compared to SD control confirmed diastolic dysfunction in males fed with the VD and AD respectively. Among females, only the AD group showed an increase of this ratio compared to control (Table 2), and that by 90%. For its part, LV mass was significantly increased in the mice fed with VD and AD (24% and 18% in males and 17% and 14% in females respectively). This increase of LV mass was essentially caused by the thickness of the interventricular septum and of the LV posterior wall (Table 2). Interestingly, the LV hypertrophy in the VD female group was not associated with diastolic-dysfunction as observed in the other HFD groups.

**Table 2:** Evaluation of cardiac parameters by Doppler echography

Sex groups	Diet groups	E/A ratio ± SEM	LV mass (mg) ± SEM	Inter-ventricular septum (mm) ± SEM	LV posterior wall (mm) ± SEM
**Male** **mice**	SD	1.39 ± 0.15	109.8 ± 2.6	0.72 ± 0.01	0.76 ± 0.02
	
	VD	2.27 ± 0.14***	135.7 ± 7.5 **	0.83 ± 0.03 **	0.85 ± 0.02 **
	
	AD	2.20 ± 0.08***	129.8 ± 6.2 **	0.89 ± 0.02 ***	0.84 ± 0.01 *

	
**Female** **mice**	SD	1.56 ± 0.05	101.1 ± 2.7	0.77 ± 0.01	0.70 ± 0.02
	
	VD	1.62 ± 0.08	118.7 ± 5.0 *	0.86 ± 0.03 **	0.78 ± 0.02 *
	
	AD	3.01 ± 0.45***^###^	115.7 ± 3.6 *	0.85 ± 0.03 *	0.79 ± 0.01 **

The early wave (E) and the late wave (A) of the mitral flow. LV mass was calculated by the measurement of the inter-ventricular septum, posterior wall thicknesses, and the tele-diastolic diameter. * P < 0.05, ** P < 0.01, *** P < 0.001 vs. SD; ### P < 0.001 vs. VD.

## Discussion

HFD-induced obesity has become widely accepted as a key factor of alteration in insulin sensitivity and metabolism [9,11]. However, the physiological regulation and role of HFD in mediating the unhealthy effects of increased adiposity remain not fully elucidated.

The novelty of this study is the use of two types of HFD identical in their lipid proportion but different in their fat nature. These diets were low in cholesterol in order to reduce its implication in metabolic alterations and mimic industrially-produced popular fast food known as obesity and T2D inducers [9,11,19]. Both HFD promoted more weight gain than the control SD with a faster increase in males compared to females. However, the degrees of metabolic alterations differed considerably between the two HFD and were highly influenced by gender. This was not a consequence of the daily caloric uptake but was rather due to the dietary fat nature and mice gender.

For a comparable weight gain in HFD groups, males and females under AD showed a more pronounced accumulation of adipose tissue in the abdominal visceral region compared to corresponding VD groups. Abdominal visceral adipose tissue mass was slightly increased in VD group compared to control but remained non significant when reported as percentage of total body weight. Thus, despite its implication in increasing weight gain, a VD promotes a fat mass distribution different than an AD in both genders. In fact, it is well established that mono-unsaturated FA are more accumulated in the subcutaneous region and prevent central fatty acid accumulation [20]. Furthermore, it was reported that postprandial fat oxidation as well as a meal thermic effect was higher with mono-unsaturated FA rich diet compared to a saturated FA rich diet [21]. The increased thermic effect could reflect a pronounced storage activity of the subcutaneous adipose mass. Thus, we can speculate that our mono-unsaturated FA rich diet trigger a different adipose tissue accumulation than the saturated FA rich AD. However, more experiences are needed to confirm this hypothesis.

This differential fat accumulation plays a crucial role in metabolic alterations development. A link was established between HOMA-IR increase and the visceral adipose tissue accumulation in AD-fed groups. These results support previous findings that demonstrate a correlation between visceral abdominal adipose tissue accumulation and metabolic alterations; a correlation not reported for subcutaneous fat accumulation [22,23]. High concentrations of saturated FA such as in AD are associated with lipotoxicity effects (pancreas, liver, muscle, adipose tissue...) and alter cell membrane dynamics [24]. This prevents the dimerization of cell surface receptors such as insulin and leptin receptors and inhibits the signalization of the corresponding hormone. Therefore, the increased levels of insulin and leptin in our model could reflect a defect in their respective signalling pathway. In addition, increased insulin level and HOMA-IR are obtained simultaneously with enhanced resistin levels in AD groups. Resistin secretion in adipose tissue is known to induce insulin resistance through the inhibition of its signalling pathway and the stimulation of hepatic glucose production [25]. These data could explain the fact that AD mice developed increased insulin circulating levels. The VD, rich in unsaturated FA, did not affect insulin levels nor modulates resistin levels in corresponding groups as reported in a previous study with unsaturated oleic acid [26]. So, at similar circulating levels, different combinations of FA promote distinctive alterations in the systemic glucose homeostasis.

Female mice remained normo-glycemic under HFD confirming less advancement in metabolic alterations compared to males. This female-specific resistance to T2D development was even more striking with VD compared to AD, which induced hyper-insulinæmia and slight glucose intolerance. However, VD as well as AD induced T2D with an increased fasting glycæmia and glucose intolerance in male mice and that starting the 12^th ^week of HFD feeding. These alterations could be correlated with adipocytes death demonstrated in HFD-fed male mice [27]. This parameter was however never been investigated in HFD-fed female mice. On the other hand, our preliminary study didn't show any increase in the glycaemia of the HFD-fed female mice even after 16 weeks of diet. We have thus extended our protocol to 20 weeks. It is well established that females show less extensive metabolic alterations due to the oestrogen action that protect adipocytes in female from insulin resistance and inflammation [12,13,28]. In our model, we have demonstrated that females had a higher level of ERα mRNA in their adipocytes. Its mRNA expression level could reflect a higher activity of ERα in corresponding tissue. Furthermore, recent evidence shows that FA accumulation is more oriented to the visceral fat pad in male while it is more directed to the peripheral region [29]. Thus, since android fat accumulation is more associated to metabolic alterations development [22], these observations could explain the prevention of T2D development in female groups.

Both HFD increased leptin levels in male and female mice with a significant difference between VD and AD groups. Correlated with body weight increase, high circulating leptin levels, known to improve glucose homeostasis [30], suggests leptin resistance in obese mice. Leptin resistance affects glucose homeostasis and contributes directly to hyperglycaemia [30,31]. Male mice showed increased leptin levels concurrently with increased AUC values of the IPGTT. In female groups, the apparent lack of negative leptin action on glycaemia could be due to a counterbalanced effect by the high adiponectin levels compared to male groups. Adiponectin, an insulin-sensitizer adipokine and an inhibitor of hepatic glucose production, contributes to improved glucose homeostasis [32,33].

To evaluate the direct impact of diets on adipose tissue, additional analyses were performed at the mRNA level. TNFα is a key regulator of the adipogenesis that decreases insulin sensitivity and promotes free FA production by stimulating lipolysis and inhibiting the antilipolytic effects of insulin [34,35]. In our model, TNFα mRNA levels in visceral abdominal adipose tissue were increased in males under AD and, to a much lesser extent, in VD-fed males. This effect could be due to enhanced macrophage infiltration in the adipose tissue [27,35,36]. In the AD group, it was associated with leptin and resistin circulating levels known to augment TNFα production [37,38]. In the VD group, oleic acid may have contributed to limit TNFα up-regulation. This FA was found to be effective in reversing the inflammatory status in adipose tissue responsible for decreased insulin sensitivity [36]. In the AD female group, despite their increased levels of leptin and resistin, TNFα mRNA levels remained unchanged in the visceral adipose tissue. A high level of circulating adiponectin is known to inhibit TNFα effects [39,40]. Adiponectinemia in HFD-fed group was reduced in female mice compared to control. However, these levels of adiponectin seem to still be enough to prevent TNFα mRNA up regulation in the adipose tissue. Moreover, it could also have contributed to the delay of T2D development regardless of high leptin levels [40]. However, decreased mRNA expression levels of adiponectin in the adipose tissue of females under HFD could be the first sign of progression toward a metabolic alteration cascade.

Thus, adiponectin modulation seems to play a critical role in our model and could contribute to differences between males and females, with delayed metabolic alterations in the latter.

Hyperplasia and hypertrophy of adipocytes are involved in obesity [41]. The balance between the two processes is controlled by FA nature that governs nuclear receptors activity [42]. Therefore, inhibition of adipogenesis through high levels of circulating FA triggers adipocytes hypertrophy and leads to insulin resistance [43], a situation encountered in the AD mice group. In contrast with the oleic-rich VD-fed groups, metabolic alterations were less extensive with an absence of hyper-insulinæmia. In this case, adipocyte hypertrophy and hyperplasia occurred. Hyperplasic obesity is accepted as being less harmful regarding metabolic alterations and adipose tissue inflammation [35,36].

Dietary FA modulate PPARγ activity which controls adipokines secretion [43,44]. Superior adipogenesis potential in white adipose tissue of the abdominal visceral region of females over males is suggested by the pattern of expression levels of the PPARγ2 gene. Our results showed decreased levels of PPARγ2 mRNA in HFD groups and remained higher in females compared to males. The highest levels were found in the VD female group protected against HFD-mediated negative metabolic impacts. These results support the hypothesis about the role of PPARγ2 in preventing adipocytes hypertrophy that leads to decrease adiponectin circulating levels and insulin sensitivity.

In previous studies, crosstalk between the estrogens receptors and PPARγ regulatory pathways has been demonstrated with sex hormone regulation of adipokine production [16,42]. Expression of ERα, a main mediator of oestrogen effects, was investigated as it could be involved in the gender regulation and/or diet-specific response of adipose tissue. In this study, ERα mRNA levels in adipocytes were decreased with HFD but remained higher in female groups. Such higher expression levels of ERα in females may favour PPARγ2 mRNA expression under HFD when compared to males [16].

Signs of cardiac diastolic dysfunction linked to weight gain and metabolic alterations were also detected in the HFD groups. An increase of the E/A ratio, with restrictive aspects of transmitral flow (E/A > 2), indicated a diastolic dysfunction with increased LV filling pressure. A significant elevation of the E/A ratio occurred in males fed with the VD or AD (63% and 59%, respectively) and a 90% increase in the AD female group only. Females on VD had no modification of the E/A ratio despite being overweight. Obesity can be associated with impaired LV diastolic function [45] though the exact reason is still unclear. Leptin regulation of the hypothalamic-pituitary-adrenal axis responsible for blood pressure regulation [31], is disturbed in obese subjects. Thus, high leptin concentration leads to diastolic dysfunction associated with higher cardiac sympatic nervous system activity and increased LV mass [45]. This dysfunction with a reduction in cardiac compliance was associated with LV dilatation and an increased LV mass in HFD groups.

Hyperglycaemia and hyper-insulinæmia have also been suggested to be additive stimuli to LV hypertrophy [46]. Thus, the fasting hyperglycaemia in male mice under HFD and the elevated level of circulating insulin in mice under AD could have aggravated cardiac hypertrophy and alteration. In fact, in glucose diabetic and insulin resistant mice, the myocardium consumes more FA to produce energy leading to more LV hypertrophy. Female mice under VD increased their LV mass without diastolic dysfunction. The normal glycemic and insulin rates in the presence of higher adiponectin concentration compared to the male group contributed to maintain normal diastolic function. Furthermore, the VD contains 27% more oleic acid, which is known to prevent cardiac dysfunctions [47], than the AD. These differences in diet composition could explain the prevention of diastolic dysfunction in the female VD group but not in the female AD group.

Metabolic alterations development is different between males and females. However, the majority of studies on HFD-induced metabolic disorders are restricted to males [48]. The strength of this work was therefore the evaluation of the respective sensitivity of both sexes to two types of low-cholesterol HFD, differing in their dietary FA nature. Thus, we have shown that combinations of FA have gender-related effects on visceral fat distribution and metabolic consequences. Therefore, susceptibility to develop HFD-linked T2D is strongly reflected by sex hormone-associated modulation of adiponectin production, TNFα and PPARγ regulation in visceral adipose tissue. However, further investigation will be required to explain the differences in response to the two types of HFD among females.

Understanding gender-specific adipose tissue adaptations underlying metabolic disorders linked to HFD and unhealthy lifestyles will considerably contribute to the development of improved strategies for the prevention and treatment of metabolic and cardiovascular diseases.

## Conclusions

Combinations of FA have gender-related effects on visceral fat distribution and metabolic consequences. Susceptibility to develop HFD-linked T2D is strongly reflected by gender modulation of adiponectin production, TNFα and PPARγ regulation in visceral adipose tissue. The evaluation of the hormone modulations in our mice model could provide more answers on the remained unclear sex differences.

## Abbreviations

AD: Animal high fat Diet; ANCOVA: analysis of covariance; ERα: Estrogens Receptor alpha; FA: Fatty Acid; HFD: High Fat Diets; IPGTT: Intra-Peritoneal Glucose Tolerance Test; PPARγ: Peroxisome Proliferator-Activated Receptors gamma; SD: Standard Diet; T2D: Type 2 Diabetes; TNFα: Tumour Necrosis Factor alpha; VD: Vegetal high fat Diet.

## Competing interests

The authors declare that they have no competing interests.

## Authors' contributions

SEA and VL carried out the physiological follow up of the mice and assays in vivo. Molecular and biochemical studies and the drafting of the manuscript were completed by SEA. IC as well was JFT participated in the design and the coordination of the study and helped to draft the manuscript. All authors read and approved the final manuscript.

## Supplementary Material

Additional file 1 **Additional data**. This file contains a table (Additional table S1) showing the relationship between weight gain and hemodynamic parameters, a table (Additional table S2) summarizing biological parameters for all mouse groups and a figure (Additional figure S1) reporting the TNFα gene expression profile. Click here for file

## References

[B1] Schuster DP Obesity and the development of type 2 diabetes: the effects of fatty tissue inflammation Diabetes Metab Syndr Obes 3 253 262 10.2147/dmsott.s7354 PMC3047970 21437093

[B2] Ahima RS Digging deeper into obesity J Clin Invest 121 2076 2079 10.1172/JCI58719 PMC3104789 21633174

[B3] Buechler C Wanninger J Neumeier M Adiponectin, a key adipokine in obesity related liver diseases World J Gastroenterol 17 2801 2811 10.3748/wjg.v17.i23.2801 PMC3120939 21734787

[B4] Schwartz DR Lazar MA Human resistin: found in translation from mouse to man Trends Endocrinol Metab 22 259 265 10.1016/j.tem.2011.03.005 PMC3130099 21497511

[B5] Wauman J Tavernier J Leptin receptor signaling: pathways to leptin resistance Front Biosci 17 2771 2793 10.2741/3885 21622208

[B6] Wang P Mariman E Renes J Keijer J The secretory function of adipocytes in the physiology of white adipose tissue J Cell Physiol 2008 216 3 13 10.1002/jcp.21386 18264975

[B7] Unger RH Clark GO Scherer PE Orci L Lipid homeostasis, lipotoxicity and the metabolic syndrome Biochim Biophys Acta 1801 209 214 10.1016/j.bbalip.2009.10.006 19948243

[B8] Declercq V Taylor C Zahradka P Adipose tissue: the link between obesity and cardiovascular disease Cardiovasc Hematol Disord Drug Targets 2008 8 228 237 10.2174/187152908785849080 18781935

[B9] Bessesen DH Update on obesity J Clin Endocrinol Metab 2008 93 2027 2034 10.1210/jc.2008-0520 18539769

[B10] Mirza MS Obesity, Visceral Fat, and NAFLD: Querying the Role of Adipokines in the Progression of Nonalcoholic Fatty Liver Disease ISRN Gastroenterol 2011 592404 10.5402/2011/592404 PMC3168494 21991518

[B11] Shertzer HG Woods SE Krishan M Genter MB Pearson KJ Dietary whey protein lowers the risk for metabolic disease in mice fed a high-fat diet J Nutr 141 582 587 10.3945/jn.110.133736 PMC3056576 21310864

[B12] Medrikova D Jilkova ZM Bardova K Janovska P Rossmeisl M Kopecky J Sex differences during the course of diet-induced obesity in mice: adipose tissue expandability and glycemic control Int J Obes (Lond) 10.1038/ijo.2011.87 21540832

[B13] Macotela Y Boucher J Tran TT Kahn CR Sex and depot differences in adipocyte insulin sensitivity and glucose metabolism Diabetes 2009 58 803 812 10.2337/db08-1054 19136652 PMC2661589

[B14] Moussavi N Gavino V Receveur O Could the quality of dietary fat, and not just its quantity, be related to risk of obesity? Obesity (Silver Spring) 2008 16 7 15 10.1038/oby.2007.14 18223605

[B15] Funaki M Saturated fatty acids and insulin resistance J Med Invest 2009 56 88 92 10.2152/jmi.56.88 19763019

[B16] Jeong S Yoon M 17beta-Estradiol inhibition of PPARgamma-induced adipogenesis and adipocyte-specific gene expression Acta Pharmacol Sin 32 230 238 10.1038/aps.2010.198 PMC4009938 21293475

[B17] Matthews DR Hosker JP Rudenski AS Naylor BA Treacher DF Turner RC Homeostasis model assessment: insulin resistance and beta-cell function from fasting plasma glucose and insulin concentrations in man Diabetologia 1985 28 412 419 10.1007/BF00280883 3899825

[B18] Shinohara K Shoji T Emoto M Tahara H Koyama H Ishimura E Miki T Tabata T Nishizawa Y Insulin resistance as an independent predictor of cardiovascular mortality in patients with end-stage renal disease J Am Soc Nephrol 2002 13 1894 1900 10.1097/01.ASN.0000019900.87535.43 12089386

[B19] Stender S Dyerberg J Astrup A High levels of industrially produced trans fat in popular fast foods N Engl J Med 2006 354 1650 1652 10.1056/NEJMc052959 16611965

[B20] Paniagua JA Gallego de la Sacristana A Romero I Vidal-Puig A Latre JM Sanchez E Perez-Martinez P Lopez-Miranda J Perez-Jimenez F Monounsaturated fat-rich diet prevents central body fat distribution and decreases postprandial adiponectin expression induced by a carbohydrate-rich diet in insulin-resistant subjects Diabetes Care 2007 30 1717 1723 10.2337/dc06-2220 17384344

[B21] Piers LS Walker KZ Stoney RM Soares MJ O'Dea K The influence of the type of dietary fat on postprandial fat oxidation rates: monounsaturated (olive oil) vs saturated fat (cream) Int J Obes Relat Metab Disord 2002 26 814 821 12037652 10.1038/sj.ijo.0801993

[B22] Kang SM Yoon JW Ahn HY Kim SY Lee KH Shin H Choi SH Park KS Jang HC Lim S Android fat depot is more closely associated with metabolic syndrome than abdominal visceral fat in elderly people PLoS One 6 e27694 10.1371/journal.pone.0027694 PMC3214067 22096613

[B23] Saad F Gooren LJ The role of testosterone in the etiology and treatment of obesity, the metabolic syndrome, and diabetes mellitus type 2 J Obes 2011 10.1155/2011/471584 PMC2931403 20847893

[B24] Manco M Calvani M Mingrone G Effects of dietary fatty acids on insulin sensitivity and secretion Diabetes Obes Metab 2004 6 402 413 10.1111/j.1462-8902.2004.00356.x 15479216

[B25] Steppan CM Bailey ST Bhat S Brown EJ Banerjee RR Wright CM Patel HR Ahima RS Lazar MA The hormone resistin links obesity to diabetes Nature 2001 409 307 312 10.1038/35053000 11201732

[B26] Rea R Donnelly R Effects of metformin and oleic acid on adipocyte expression of resistin Diabetes Obes Metab 2006 8 105 109 10.1111/j.1463-1326.2005.00477.x 16367888

[B27] Altintas MM Rossetti MM Nayer B Puig A Zagallo P Ortega LM Johnson KB McNamara G Reiser J Mendez AJ Nayer A Apoptosis, Mastocytosis, and Diminished Adipocytokine Gene Expression Accompany Reduced Epididymal Fat Mass in Long-Standing Diet-Induced Obese Mice Lipids Health Dis 10 198 10.1186/1476-511X-10-198 PMC3229589 22051061

[B28] Stubbins RE Najjar K Holcomb VB Hong J Nunez NP Estrogen alters adipocyte biology and protects female mice from adipocyte inflammation and insulin resistance Diabetes Obes Metab10.1111/j.1463-1326.2011.01488.x PMC3236284 21834845

[B29] Koutsari C Ali AH Mundi MS Jensen MD Storage of circulating free fatty acid in adipose tissue of postabsorptive humans: quantitative measures and implications for body fat distribution Diabetes 60 2032 2040 10.2337/db11-0154 PMC3142075 21659500

[B30] Toyoshima Y Gavrilova O Yakar S Jou W Pack S Asghar Z Wheeler MB LeRoith D Leptin improves insulin resistance and hyperglycemia in a mouse model of type 2 diabetes Endocrinology 2005 146 4024 4035 10.1210/en.2005-0087 15947005

[B31] Steinberg GR Parolin ML Heigenhauser GJ Dyck DJ Leptin increases FA oxidation in lean but not obese human skeletal muscle: evidence of peripheral leptin resistance Am J Physiol Endocrinol Metab 2002 283 E187 192 12067860 10.1152/ajpendo.00542.2001

[B32] Yamauchi T Kamon J Minokoshi Y Ito Y Waki H Uchida S Yamashita S Noda M Kita S Ueki K Adiponectin stimulates glucose utilization and fatty-acid oxidation by activating AMP-activated protein kinase Nat Med 2002 8 1288 1295 10.1038/nm788 12368907

[B33] Kadowaki T Yamauchi T Adiponectin and adiponectin receptors Endocr Rev 2005 26 439 451 10.1210/er.2005-0005 15897298

[B34] Lang CH Dobrescu C Bagby GJ Tumor necrosis factor impairs insulin action on peripheral glucose disposal and hepatic glucose output Endocrinology 1992 130 43 52 10.1210/en.130.1.43 1727716

[B35] Oliver E McGillicuddy F Phillips C Toomey S Roche HM The role of inflammation and macrophage accumulation in the development of obesity-induced type 2 diabetes mellitus and the possible therapeutic effects of long-chain n-3 PUFA Proc Nutr Soc 69 232 243 10.1017/S0029665110000042 20158940

[B36] Vassiliou EK Gonzalez A Garcia C Tadros JH Chakraborty G Toney JH Oleic acid and peanut oil high in oleic acid reverse the inhibitory effect of insulin production of the inflammatory cytokine TNF-alpha both in vitro and in vivo systems Lipids Health Dis 2009 8 25 10.1186/1476-511X-8-25 19558671 PMC2706835

[B37] Rabe K Lehrke M Parhofer KG Broedl UC Adipokines and insulin resistance Mol Med 2008 14 741 751 19009016 10.2119/2008-00058.Rabe PMC2582855

[B38] Warne JP Tumour necrosis factor alpha: a key regulator of adipose tissue mass J Endocrinol 2003 177 351 355 10.1677/joe.0.1770351 12773114

[B39] Bruun JM Lihn AS Verdich C Pedersen SB Toubro S Astrup A Richelsen B Regulation of adiponectin by adipose tissue-derived cytokines: in vivo and in vitro investigations in humans Am J Physiol Endocrinol Metab 2003 285 E527 533 12736161 10.1152/ajpendo.00110.2003

[B40] Zhao T Hou M Xia M Wang Q Zhu H Xiao Y Tang Z Ma J Ling W Globular adiponectin decreases leptin-induced tumor necrosis factor-alpha expression by murine macrophages: involvement of cAMP-PKA and MAPK pathways Cell Immunol 2005 238 19 30 10.1016/j.cellimm.2005.12.002 16438946

[B41] Fernandez-Quintela A Churruca I Portillo MP The role of dietary fat in adipose tissue metabolism Public Health Nutr 2007 10 1126 1131 17903320 10.1017/S1368980007000602

[B42] Shi H Clegg DJ Sex differences in the regulation of body weight Physiol Behav 2009 97 199 204 10.1016/j.physbeh.2009.02.017 19250944 PMC4507503

[B43] Kubota N Terauchi Y Miki H Tamemoto H Yamauchi T Komeda K Satoh S Nakano R Ishii C Sugiyama T PPAR gamma mediates high-fat diet-induced adipocyte hypertrophy and insulin resistance Mol Cell 1999 4 597 609 10.1016/S1097-2765(00)80210-5 10549291

[B44] Brown JD Plutzky J Peroxisome proliferator-activated receptors as transcriptional nodal points and therapeutic targets Circulation 2007 115 518 533 10.1161/CIRCULATIONAHA.104.475673 17261671

[B45] Grassi G Seravalle G Quarti-Trevano F Dell'Oro R Arenare F Spaziani D Mancia G Sympathetic and baroreflex cardiovascular control in hypertension-related left ventricular dysfunction Hypertension 2009 53 205 209 19124679 10.1161/HYPERTENSIONAHA.108.121467

[B46] Bidasee KR Nallani K Yu Y Cocklin RR Zhang Y Wang M Dincer UD Besch HR Jr Chronic diabetes increases advanced glycation end products on cardiac ryanodine receptors/calcium-release channels Diabetes 2003 52 1825 1836 10.2337/diabetes.52.7.1825 12829653

[B47] Carrero JJ Fonolla J Marti JL Jimenez J Boza JJ Lopez-Huertas E Intake of fish oil, oleic acid, folic acid, and vitamins B-6 and E for 1 year decreases plasma C-reactive protein and reduces coronary heart disease risk factors in male patients in a cardiac rehabilitation program J Nutr 2007 137 384 390 17237316 10.1093/jn/137.2.384

[B48] Srinivasan K Ramarao P Animal models in type 2 diabetes research: an overview Indian J Med Res 2007 125 451 472 17496368

