# Supplementary material for: Nature of fatty acids in high fat diets differentially delineates obesity-linked metabolic syndrome components in male and female C57BL/6J mice
Source: Diabetol Metab Syndr. 2011 Dec 14;3:34. doi: 10.1186/1758-5996-3-34 (PMC3277487; doi:10.1186/1758-5996-3-34)
Supplement: Additional file 1 — Additional data. This file contains a table (Additional table S1) showing the relationship between weight gain and hemodynamic parameters, a table (Additional table S2) summarizing biological parameters for all mouse groups and a figure (Additional figure S1) reporting the TNFα gene expression profile. [file 1758-5996-3-34-S1.DOC]

**Additional table 1. Relationship between weight gain and hemodynamic parameters.**

|  | Leptin mRNA level (n=8) | Leptin (n=8) | Adiponectin mRNA level (n=8) | Adiponectin (n=8) |
| --- | --- | --- | --- | --- |
| Leptin (n=8) | r^*^ = 0.83  p < 0.0001 | r[[1]](#footnote-2) = 1 | --- | r^*^ = -0.35  p = 0.1647 |
| Adiponectin (n=8) | --- | r^*^ = -0.35  p = 0.1647 | r^*^ = 0.59  p = 0.0764 | r^*^ = 1 |
| Weight gain (n=8) | r^*^ = -0.93  p < 0.0001 | r* = 0.88  p < 0.0001 | r^*^ = -0.73  p < 0.01 | r^*^ = -0.63  p = 0.0918 |
| Glycaemia (n=8) | r^*^ = 0.27  p = 0.0872 | r* = 0.13  p = 0.5060 | r^*^ = -0.35  p < 0.05 | r* = -0.22  p = 0.2946 |

**Additional table 2 Biological parameters for all mouse groups.**

|  | **SD** | |  | **VD** | |  | **AD** | |
| --- | --- | --- | --- | --- | --- | --- | --- | --- |
|  | **Male** | **Female** |  | **Male** | **Female** |  | **Male** | **Female** |
| BW_0_ (g) | 18.60±0.50 | 15.70±0.40^a^ |  | 18.70±0.40 | 16.10±0.20^a^ |  | 17.10±0.30 | 16.07±0.20 |
| BW_20_ (g) | 30.31±1.03 | 21.98±0.24^a^ |  | 46.83±1.37^b^ | 34.69±0.69^a,b^ |  | 40.34±0.89^b,c^ | 36.40±1.16^b^ |
| BWG (g) | 11.84±0.92 | 6.00±0.20^a^ |  | 28.08±1.25^b^ | 18.70±0.82^a,b^ |  | 23.88±0.61^b^ | 20.52±1.17^b^ |
| FI (g/day) | 3.50±0.05 | 3.60±0.09 |  | 2.80±0.17^b^ | 2.70±0.76^b^ |  | 2.40±0.12^b^ | 2.30±0.51^b^ |
| FI (kCal/day) | 10.15±0.10 | 10.44±0.29 |  | 12.88±0.60 | 12.42±2.76 |  | 12.48±0.5 | 11.96±2.60 |
| vWAT (g) | 3.90±0.14 | 2.30±0.11 |  | 8.40±0.88^b^ | 4.90±0.15^a,b^ |  | 15.20±1.15^b,c^ | 11.20±0.49^a,b,c^ |
| vWAT (% of BW_20_) | 12.94±0.71 | 10.61±0.60 |  | 17.83±1.56 | 14.03±0.50^a^ |  | 37.40±2.35^b,c^ | 26.00±1.05^a,b,c^ |

Abbreviations: BW_0_ = body weight at the start of the protocol; BW_20_ = body weight at the end of the protocol after 20 weeks of diet; BWG = body weight gain between the beginning and the end of the protocol; FI = food intake; vWAT, visceral white adipose tissue weight. a = significant differences (P<0.05) between sexes within diet; b = significant differences (P<0.05) between diets within sex compared to SD group; c = significant differences (P<0.05) between diets within sex compared to VD group. Data are shown as means±SEM.

**Additional figure 1. TNFα gene expression profile.** TNFα mRNA expression levels in abdominal visceral white adipose tissue extracted from male and female (n=8) of SD, VD and AD mice groups. mRNA expression level was evaluated by Q-PCR and reported as a ratio over the expression level of the reference gene Cyclophilin-A. *P<0.05, ***P<0.001 vs. SD; ###P<0.001 vs. VD.


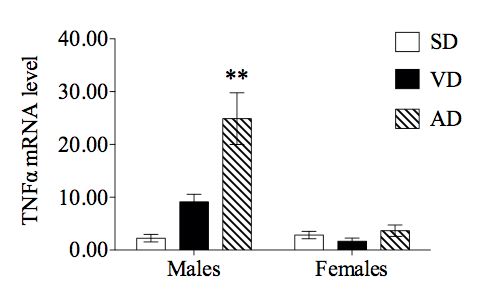


1. Pearson or Spearman correlations depending distribution of the data [↑](#footnote-ref-2)
